# High‐altitude adaptation in vertebrates as revealed by mitochondrial genome analyses

**DOI:** 10.1002/ece3.8189

**Published:** 2021-10-05

**Authors:** Xibao Wang, Shengyang Zhou, Xiaoyang Wu, Qinguo Wei, Yongquan Shang, Guolei Sun, Xuesong Mei, Yuehuan Dong, Weilai Sha, Honghai Zhang

**Affiliations:** ^1^ College of Life Science Qufu Normal University Qufu China

**Keywords:** evolution, low atmospheric O_2_ levels, mtDNA protein‐coding genes, vertebrate

## Abstract

The high‐altitude environment may drive vertebrate evolution in a certain way, and vertebrates living in different altitude environments might have different energy requirements. We hypothesized that the high‐altitude environment might impose different influences on vertebrate mitochondrial genomes (mtDNA). We used selection pressure analyses and PIC (phylogenetic independent contrasts) analysis to detect the evolutionary rate of vertebrate mtDNA protein‐coding genes (PCGs) from different altitudes. The results showed that the ratio of nonsynonymous/synonymous substitutions (*dN/dS*) in the mtDNA PCGs was significantly higher in high‐altitude vertebrates than in low‐altitude vertebrates. The seven rapidly evolving genes were shared by the high‐altitude vertebrates, and only one positive selection gene (*ND5 *gene) was detected in the high‐altitude vertebrates. Our results suggest the mtDNA evolutionary rate in high‐altitude vertebrates was higher than in low‐altitude vertebrates as their evolution requires more energy in a high‐altitude environment. Our study demonstrates the high‐altitude environment (low atmospheric O_2_ levels) drives vertebrate evolution in mtDNA PCGs.

## Graphical abstract

We studied the influence of different altitudes on the evolution of 104 vertebrate mtDNA PCGs for the first time. Our results demonstrated that the rapid evolution and positive selection sites of mitochondrial genes are one of the important factors for vertebrates to adapt to plateau environment through this research and the high‐altitude environment drove the evolution of vertebrate mtDNA PCGs.

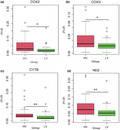

## 1 INTRODUCTION

Due to the distinctive geographical environment, high altitudes involve severe environmental conditions, such as low pressure, low oxygen, and high UV (ultraviolet) radiation. Low oxygen is one of the most important factors affecting vertebrate survival. Vertebrates adapt to hypoxia in high‐altitude habitats through changes in their physiological phenotype and gene evolution (McCracken et al., 2009; Zhu et al., 2018). High‐altitude vertebrates represent a unique scenario for studying adaptation. Therefore, studying adaptation in high‐altitude vertebrates is of great significance in allowing us to explore the adaptive evolution of organisms.

Mitochondrion energy metabolism plays an important role in the adaptation to the plateau environment by vertebrates through aerobic respiration (Das, 2006; Gershoni et al., 2009). The adenosine triphosphate (ATP) produced by mitochondrion can maintain vertebrate body temperatures and energy for activities (Sun et al., 2011). The mitochondrial genome contains thirteen protein‐coding genes (mtDNA PCG) encoding thirteen proteins involved in aerobic respiration (Chong & Mueller, 2013). To adapt to hypoxia environments, aerobic respiration in high‐altitude vertebrates may undergo natural selection to increase efficiency. Therefore, the mitochondrial evolutionary rate may be affected by the low atmospheric O2 levels at high altitudes (Scott et al., 2009).

At present, several studies using mitochondrial genome analyses have demonstrated that the thirteen animal mtDNA PCGs have different environmental adaptations based on different evolutionary rates and selection constraints, including mammals (Gu et al., 2012; Luo et al., 2008), aves (Scott et al., 2009; Zhou et al., 2014), and actinopterygii (Li et al., 2013; Wang et al., 2016). For example, the NS/S values of the *ATP6*, *ATP8*, and *Cytb* genes were larger (>1) in the Tibetan than the Han population (Gu et al., 2012). Similarly, the bar‐headed geese had a striking alteration in the kinetics of the cytochrome c oxidase (COX) in high‐altitude environments (Scott et al., 2009). Wang et al. (2016) discovered that Tibetan loaches accumulated more nonsynonymous mutations and exhibited rapid evolution when compared to non‐Tibetan loaches. It is possible that the mtDNA of low‐altitude vertebrates might have a lower evolutionary rate. In contrast, the mtDNA of high‐altitude vertebrates may have a faster evolutionary rate as they adapt to the low oxygen environment to maintain an efficient energy metabolism. Therefore, we tested the rate of mtDNA evolution between vertebrates living at different altitudes using the rate ratio of nonsynonymous/synonymous nucleotide substitutions (d*N*/d*S* (*ω*)). We aimed to determine whether there is a molecular basis for high‐altitude adaptation that is related to vertebrate mitochondrial evolutionary rates. This adaptation to low atmospheric O_2_ levels shows the mtDNA evolution is a critical molecular process enabling the survival of vertebrates at high altitudes and hypoxic environments driving the evolution of vertebrate mitochondrial genes.

## 2 MATERIALS AND METHODS

### 2.1 Species sample and mtDNA sequence data

Based on previous studies and the existing mitochondrial genome data of NCBI, we selected 104 vertebrate species (with habitats at varying altitudes; high‐altitude vertebrate, 52; low‐altitude vertebrate, 52). We downloaded mitochondrial genomes from the NCBI GenBank database (http://www.ncbi.nlm.nih.gov/). Our initial criterion was to only select species with clear information available on altitude. If there was no clear altitude information, our second criteria were to include species specific to the plateau. The third criteria were to select the low‐altitude species related to the high‐altitude species. The data set covered five taxa (mammal, aves, reptile, amphibian, and actinopterygii), and the data set of each taxon was greater than or equal to 20 mitochondrial genomes. For most families investigated within each class (mammal, aves, reptile, amphibian, and actinopterygii) in this study, more than one representative of each species was selected. The species and mitochondrial genome accession numbers are listed in Appendix S1.

The high‐altitude vertebrate habitat is over 2000 m, and the low‐altitude vertebrate habitat is below 1000 m. According to the habitat altitude and vertebrate phylogenetic relationships, the 104 vertebrate species were divided into 5 high‐altitude groups (high‐altitude mammal (MH), high‐altitude aves (BH), high‐altitude amphibian (AH), high‐altitude reptile (RH), and high‐altitude actinopterygii (FH)) and 5 low‐altitude groups (low‐altitude mammal (ML), low‐altitude aves (BL), low‐altitude reptile (RL), low‐altitude amphibian (AL), and low‐high actinopterygii (FL)), respectively.

### 2.2 Phylogenetic construction

We retrieved the 13 mtDNA PCGs of the mitochondrial genomes from the mtDNA sequence data and aligned the mtDNA PCGs using MUSCLE v3.8.31 (Edgar, 2004). We obtained fourteen sequence datasets (Appendix S1). The phylogenetic relationships of the 104 vertebrate species were determined using 13 mtDNA PCGs datasets and their Bayesian inference (BI). We used Mega 7.0 to assess the base substitution saturation in the 13 mtDNA PCGs datasets and used DAMBE 6.4 (Xia, 2001) to calculate the I_ss_ and I_ss.c_ for testing the substitution saturation. The optimal model (GTI +T + G) was selected in the modelfinder function of PhyloSuite software (Zhang et al., 2020) based on the greedy search algorithm and the Bayesian information criterion. The concatenated matrix was performed with four independent Markov Chain Monte Carlo (MCMC) chains for 2,000,000 generations and sampling one tree every 1000 generations.

### 2.3 Selection pressure analyses

We used the one ratio (M0) model and the free ratio model (model = 1) to estimate the ratio of nonsynonymous (d*N*) to synonymous (d*S*) substitutions rates (*ω* = *dN/dS*) using the CodeML program in PAML version 4 (Yang, 2007). The likelihood ratio tests (LRTs) were used to determine which mtDNA PCG free ratio model was not effective models. The free ratio model allowed different ω values for each branch on a tree. The *ω* values of the 13 mtDNA PCGs from the 104 species and each mtDNA PCG with their mitochondrial genomes were computed separately for the terminal branches to evaluate the selective pressure. If the d*N* or d*S* values were equal to zero, the *ω* values could be smaller or larger and we did not use these *ω* values in our analysis, and we assigned these *ω* values as NA data in the ω dataset. The *ω* values from the 5 high‐altitude groups (MH, BH, RH, AH, and FH) and 5 low‐altitude groups (ML, BL, RL, AL, and FL) were compared using one‐way ANOVA and multiple comparisons analysis. We used false discovery rates (FDR) to correct the p values. We used a Wilcoxon test to evaluate the statistical significance in the differences in *ω* values of 13 mtPCGs and each mtPSG between the species at different altitudes (high‐altitude vertebrate, HV; low‐altitude vertebrate, LV).

**FIGURE 1:**
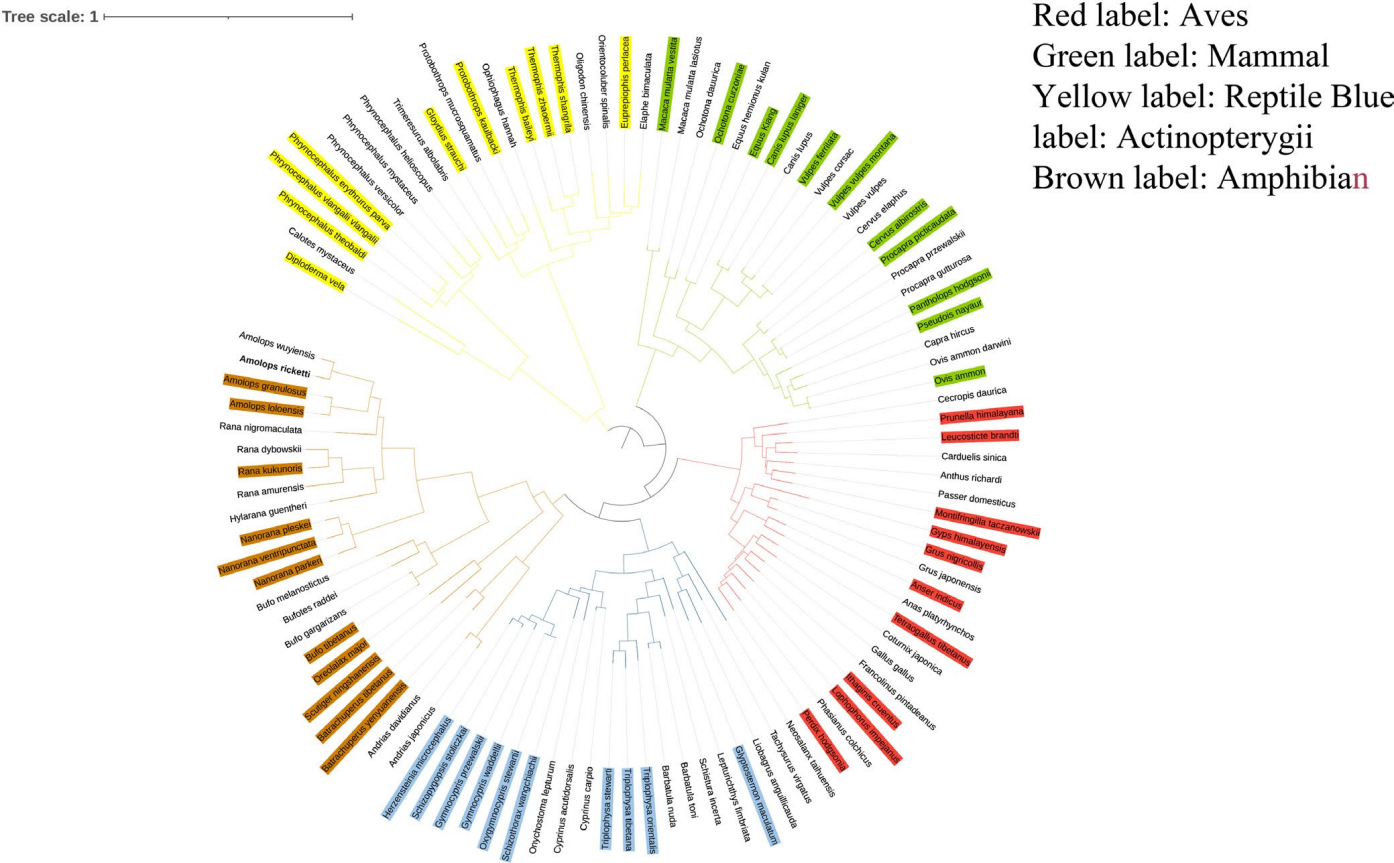
The BI phylogenetic tree of 104 vertebrates based on 13 PCGs of mitochondrial genomes (red background, high‐altitude aves; green background, high‐altitude mammal; yellow background, reptile; blue background, high‐altitude actinopterygii; brown background, high‐altitude amphibian)

To identify the positive selection gene or rapidly evolving gene for high‐altitude adaptation, we used a branch model (one ratio (M0) model, two ratio (M2) model, and NSsites = 0) to detect each mtDNA PCGs in the 104 species. In our research, the branches with high‐altitude vertebrates were used as foreground branches, and the low‐altitude vertebrates were the background branches. Furthermore, we used a branch‐site model (one ratio (M0) model, two ratio (M2) model, and NSsites = 2) in PAML to detect each mtDNA PCG of the 104 species (foreground branches, HV; background branches, LV). We used LRTs to determine which mtDNA PCGs were positive selection genes or rapidly evolving genes. These analyses were based on the BI tree (Figure 1).

### 2.4 Phylogenetic independent contrasts analysis

The closely related species (shared inheritance) may affect the species comparative analysis. Therefore, we used the phylogenetic independent contrasts (PIC) analysis (Felsenstein Joseph, 1985) to remove the closely related species influences and explore the relationship between altitude and the *ω* values of the 13 mtDNA PCGs using the ape package in R software. Firstly, we used FigTree (v1.4.3) to transform the BI tree into a binary tree. The binary tree was used as the input file in the analysis. Secondly, we classified the 104 species into two groups (high‐altitude and low‐altitude). The high‐altitude group and low‐altitude groups were coded 0 and 1, respectively. We used the *ω* values, 0 and 1, as the character data in the PIC analysis.

## 3 RESULTS

### 3.1 Phylogenetic analyses

We used 13 mtDNA PCGs datasets of the 104 vertebrates to conduct the phylogenetic analysis. The BI phylogenetic analyses of the concatenated datasets yielded consistent topological relationships between vertebrates with high bootstrap support values and Bayesian posterior probabilities. The BI tree is divided into 5 large branches: the mammal, aves, reptile, amphibian, and actinopterygii (Figure 1). Our results are consistent with previous studies (Townsend et al., 2008), and this was credible for the following analyses.

### 3.2 Free ratio model analysis

The *ω* values (13 mtDNA PCGs and each mtDNA PCG of 104 vertebrates) were estimated using CodeML within the PAML package. The *ω* values <1 indicate a purifying selection, the *ω* values = 1 indicate a neutral selection, and the *ω* values >1 indicate a positive selection. We found that the *ω* values were lower than 1 for the 13 mtDNA PCGs or each mtDNA PCG within the 104 vertebrates (provided in Appendix S2). On the other hand, we found that the *ATP8* gene experienced positive selection in the *Phrynocephalus theobaldi* (*ω* = 1.209; group RH) and *Protobothrops mucrosquamatus* lineage (*ω* = 1.132; group RL); the *COX1* gene (*ω* = 1.373) and *ND5* gene (*ω* = 1.609) experienced positive selection in the *Cyprinus acutidorsalis* lineage (group FL); and the *ND6* gene experienced positive selection in the *Barbatula toni* lineage (*ω* = 1.522; Group FH) and *Anser indicus* lineage (*ω* = 1.998; Group BH).

**FIGURE 2:**
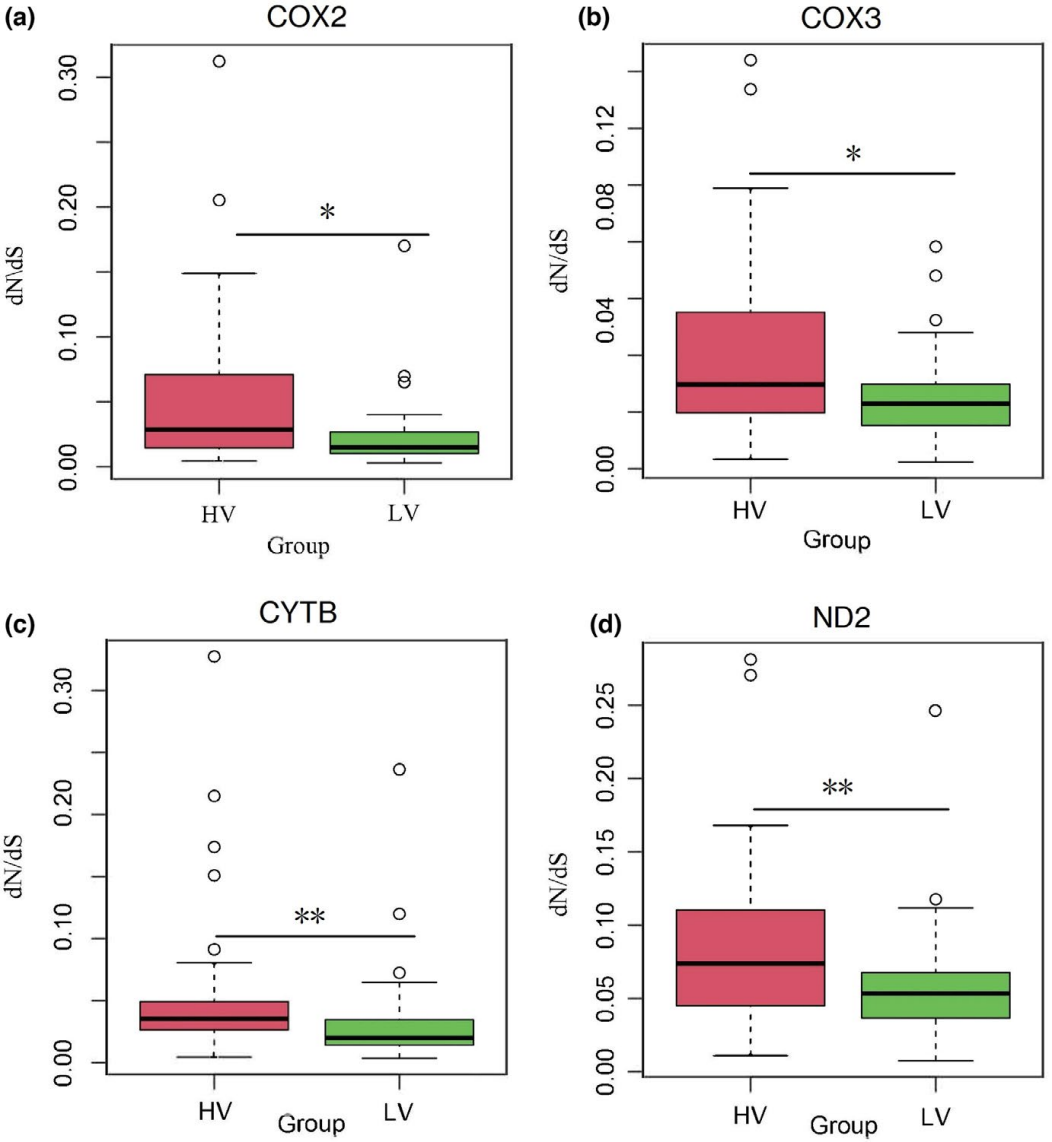
Comparisons of *ω* values among vertebrates of different altitudes based on 13 mtDNA PCGs (*p < .05; **p < .01)

The Wilcoxon test results showed that the *ω* values of the *COX2*, *COX3*, *Cytb*, and *ND2* genes were higher in the HV than the LV (Figure 2). The other mtDNA PCGs and the 13 mtDNA PCGs were not higher in the HV than the LV (*p* > .05). In addition, the one‐way ANOVA and multiple comparison analysis showed the 13 mtDNA PCGs and each mtDNA PCG were not significantly different between the 5 high‐altitude groups or the 5 low‐altitude groups (FDR *p* > .05). The results indicated that the evolutionary rates were not significantly different in the 13 mtDNA PCGs and within each mtDNA PCG in the high‐altitude vertebrates or the low‐altitude vertebrates.

### 3.3 Positive selection analysis

We used the branch model and branch‐site model analyses to test our hypothesis. The branch model analysis identified seven rapid evolutionary genes (LRTs, *p* < .05) in the HV, that is, the *ND1* gene (HV: *ω* values, 0.0351; LV: *ω* values, 0.0274), *ND2* gene (HV: *ω* values, 0.0735; LV: *ω* values, 0.0561), *ND4* gene (HV: *ω* values, 0.0533; LV: *ω* values, 0.0437), *COX2* gene (HV: *ω* values, 0.0306; LV: *ω* values, 0.0216), *COX3* gene (HV: *ω* values, 0.0287; LV: *ω* values, 0.0215), *ATP6* gene (HV: *ω* values, 0.0524; LV: *ω* values, 0.0393), and *Cytb* gene (HV: *ω* values, 0.0376; LV: *ω* values, 0.0255) (Table 1). The results confirmed the effectiveness of the Wilcoxon test results. We used a branch‐site model to detect the positive site of each mtDNA PCG in the HV. We found that only one positive selection gene (*ND5* gene, corresponding to site 247 and 524) was detected in the HV using the branch‐site model analysis (Table 2).

**TABLE 1:** The rapidly evolving gene on vertebrates 13 mtDNA PCGs through branch model

Gene	2ΔlnL	*p*‐value	The *ω* values of high‐altitude vertebrates (HV)	The *ω* values of low‐altitude vertebrates (LV)
ATP6	7.976	.004	0.0524	0.0393
ATP8	0.098	.754	0.1530	0.1611
ND1	7.763	.005	0.0351	0.0274
ND2	13.558	.0002	0.0735	0.0561
ND3	0.472	.492	0.0543	0.0496
ND4	8.084	.004	0.0533	0.0437
ND4L	0.048	.826	0.0395	0.0409
ND5	0.132	.890	0.0603	0.0571
ND6	0.109	.740	0.0556	0.0581
COX1	0.029	.863	0.0107	0.0104
COX2	9.366	.002	0.0306	0.0216
COX3	6.302	.012	0.0287	0.0215
Cytb	22.069	2.63E−06	0.0376	0.0255

**TABLE 2:** Positive selection on 13 mtDNA PCGs of mammal, aves, reptile, amphibian, and actinopterygii through branch‐site model

Models	Parameter estimates					*p*‐value	Positively selected sites (BEB analysis)
	Site class	0	1	2a	2b		
Null model	Proportion	0.94622	0.01479	0.03838	0.00060		247 F 0.952*
	Background *ω*	0.05529	1.00000	0.05529	1.00000	3.86E−14	524 M 0.991**
	Foreground *ω*	0.05529	1.00000	1.00000	1.00000		
Model A	Proportion	0.98147	0.01853	0.00000	0.00000		
	Background *ω*	0.05720	1.00000	0.05720	1.00000		
	Foreground *ω*	0.05720	1.00000	20.80002	20.80002		

Note

*represents the significant level; PPs of Bayes Empirical Bayes (BEB) analysis with *p* > .95 was regarded as candidates for selection (*> 0.95, **> 0.99)

### 3.4 Phylogenetic independent contrasts analysis

**FIGURE 3:**
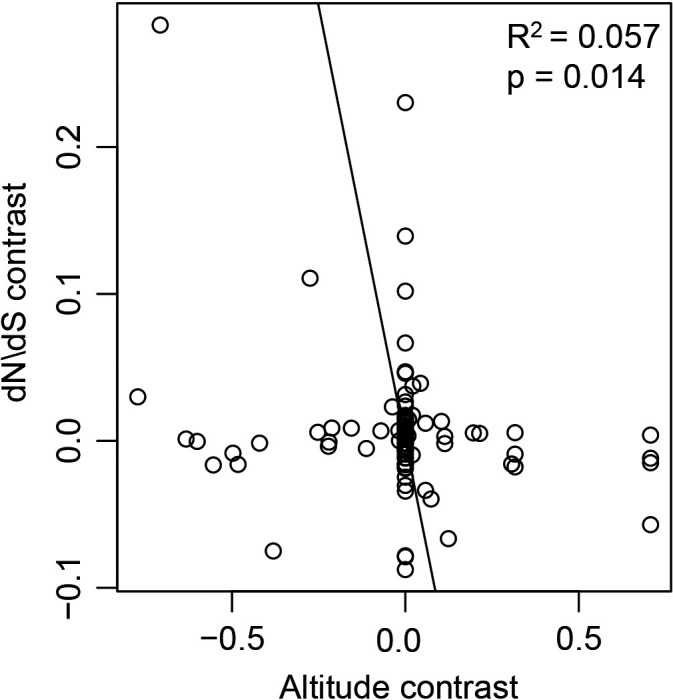
PIC analysis between different altitude and *ω* values of 104 vertebrates mitochondrial 13 mtDNA PCGs

We used a PIC analysis to overcome the potential phylogenetic biases, for example, variation in the branch lengths and any phylogenetic inertia. The PIC showed a significant decreasing trend in the d*N*/d*S* ratio with elevation reduction (*R* ^2^ = 0.057, *p* = .014; Figure 3). We identified the evolutionary rate of the 13 mtDNA PCGs were faster in the high‐altitude vertebrates when compared with low‐altitude vertebrates.

## 4 DISCUSSION

This research focused on the adaptation of vertebrate mtDNA PCGs to high‐altitude environments. In high‐altitude environments, vertebrates must maintain normal energy production under hypoxic pressure (Qiu et al., 2012; Wang et al., 2011). Therefore, high‐altitude vertebrates require more energy than low‐altitude vertebrates. Mitochondria (as the energy metabolism center of cells) provide energy for the animals through oxidative phosphorylation (Maes et al., 2004). Therefore, vertebrate mitochondria play important roles in high‐altitude adaptation. Previous research has mainly focused on one kind or family of vertebrates, such as Tibetans (Chen et al., 2020), Tibetan pigs (Ma et al., 2019), Antilopinae (Hassanin et al., 2009), Galliform birds (Zhou et al., 2014), and Schizothoracine fishes (Li et al., 2013). However, previous research has not focused on different altitudes when assessing the five vertebrate taxa. Therefore, we studied the evolutionary rate of 13 mtDNA PCGs within vertebrates at different altitudes. Our research revealed a consistent role of vertebrate mitochondria in high‐altitude adaptation.

### 4.1 Ratio of nonsynonymous/synonymous nucleotide substitutions

The ratio of nonsynonymous/synonymous nucleotide substitutions (i.e., d*N*/d*S* ratio or *ω* value) has been widely used to measure the selection pressure intensity of the protein‐coding genes (Andrieux & Arenales, 2014; Xia et al., 2019). In our research, the *ω* values of the 13 mtDNA PCGs and each mtDNA PCG in 104 vertebrates were generally lower than 1 (Appendix S2). The results indicated that the PCGs of the vertebrate mtDNA were under purifying selection at the different altitudes. Previous research has demonstrated that the mtDNA PCGs of Mustelidae (Wei et al., 2020), Tibetan loaches (Wang et al., 2016), and Orthoptera insects (Chang et al., 2020) were under purifying selection during their evolution. This relates to the highly conserved mitochondrial DNA protein‐coding genes (Wolstenholme, 1992). Purifying selection can delete deleterious substitutions to maintain the normal function of the protein‐coding genes. The *ω* values of 13 mtDNA PCGs and each mtDNA PCG were generally high among vertebrates in high altitudes. This result was consistent with previous research (Wang et al., 2016; Xu et al., 2007) and indicates that high‐altitude environments affect the evolutionary rate of vertebrate mitochondrial DNA protein‐coding genes.

Furthermore, our research also investigated the signatures of positive selection in the *ATP8* gene experienced positive selection in the linage *Phrynocephalus theobaldi* and *Protobothrops mucrosquamatus*; the *COX1* gene and *ND5* gene experienced positive selection in the *Cyprinus acutidorsalis* lineage; and the *ND6* gene experienced positive selection in the *Barbatula toni* lineage and *Anser indicus* lineage. These evolutionary events showed that the habitat of some low‐altitude vertebrates and high altitudes can affect the evolution rate of mitochondria. For example, the *Cyprinus acutidorsalis* is a specific brackish water species (Liu et al., 2004), and the special brackish water environment may drive the positive selection in the *Cyprinus acutidorsalis* mtPCGs. Therefore, to verify the effect of altitude on the evolutionary rate of vertebrate mitochondria, we used the Wilcoxon test (based on the *ω* value of the terminal branches), a branch model, a branch‐site model, and PIC analysis.

### 4.2 Selection pressure comparison

We used the Wilcoxon test, a branch model, and a branch‐site model to detect any rapidly evolving genes and positive selection genes shared among the five high‐altitude taxa. We used the Wilcoxon test and one‐way ANOVA analysis (*ω* values of 13 mtDNA PCGs and each mtDNA PCG in the terminal branches) to detect the evolutionary rate variations between the vertebrates at different altitudes and the evolutionary rate differences between different taxonomies at the same altitude (i.e., the evolutionary rate differences among mammal, aves, reptile, amphibian, and actinopterygii at high or low altitudes), respectively. The *ω* values (13 mtPCGs and each PCG) showed no significant difference between the five taxonomies at high‐ or low‐altitude environments. This may be due to the similar selection pressures (oxygen levels). The *ω* values of the *COX2* gene, *COX3* gene, *ND2* gene, and *Cytb* gene suggested they were all rapidly evolving in the high‐altitude vertebrates. These results show the high‐altitude environment (hypoxia) affects the evolutionary rate of the mitochondria in vertebrates, and the partial gene evolutionary rate in HV was faster than LV (Cheviron & Brumfield, 2012; Storz & Scott, 2019; Storz et al., 2010).

The rapidly evolving genes of the *ND1* gene, *ND2* gene, *ND4* gene, *COX2* gene, *COX3* gene, *ATP6* gene, and *Cytb* gene were detected in the high‐altitude vertebrates using the branch model. The results of the branch model contained the Wilcoxon test results. These results indicated that the rapidly evolving genes of the high‐altitude vertebrates might go through similar evolutionary patterns (Li et al., 2013; Wang et al., 2016). The high‐altitude environment drives the evolution of vertebrate mitochondrial genes (Ghezzi & Zeviani, 2012; Lenaz et al., 2006; Qingqing et al., 2018).

We detected only one positive selection gene (*ND5* gene) using the branch‐site model. The *ND5* gene is involved in the regulation of electron transfer in the respiratory chain, which provides necessary energy for vertebrate activity (Brandt, 2006; Javadov et al., 2021; Sousa et al., 2018). We suggest that the *ND5* gene is responsible for high‐altitude adaptation in vertebrate. These results suggested that high‐altitude vertebrates probably employ the same genic toolkit to adapt to the extreme environment.

### 4.3 Phylogenetic independent contrasts analysis

A PIC analysis can remove any influence by phylogenetic inertia, and we identified a significant relationship between the *ω* values of vertebrate mtDNA PCGs and habitat altitude using a regression analysis (*R* ^2^ = 0.057, *p* = .014). The *ω* values were negatively related to habitat altitude (the *ω* value decreases as the altitude decreased). This result was consistent with our previous results using the Wilcoxon test, branch model, and branch‐site model. These results show the evolution of vertebrate mtDNA PCGs is driven by high‐altitude environments.

## 5 CONCLUSION

In summary, we investigated the evolution of vertebrate mtDNA PCGs at different altitudes. The *ω* value for the 13 mtDNA PCGs was higher in high‐altitude vertebrates than in the low‐altitude vertebrates as analyzed by the Wilcoxon test, branch model, branch‐site model, and PIC analysis. Although mitochondrial evolution rates are not consistent across different vertebrate taxonomies, the *ND1* gene, *ND2* gene, *ND4* gene, *COX2* gene, *COX3* gene, *ATP6* gene, and *Cytb* gene are rapidly evolving genes that are shared among high‐altitude vertebrates. Moreover, the high‐altitude vertebrates possess one positive selection gene. The rapid evolution and positive selection sites of the mitochondrial genes are important factors in vertebrate adaptation to the plateau environment, and high‐altitude environments drive the evolution of vertebrate mtDNA PCGs.

## CONFLICT OF INTEREST

No potential conflict of interest was reported by the authors.

## AUTHOR CONTRIBUTIONS

**Xibao Wang:** Data curation (equal); Formal analysis (equal); Project administration (equal); Writing‐original draft (equal); Writing‐review & editing (equal). **Shengyang Zhou:** Formal analysis (equal). **Xiaoyang Wu:** Project administration (supporting). **Qinguo Wei:** Project administration (supporting). **Yongquan Shang:** Formal analysis (supporting). **Guolei Sun:** Formal analysis (supporting). **Xuesong Mei:** Formal analysis (supporting). **Yuehuan Dong:** Formal analysis (supporting). **Weilai Sha:** Project administration (supporting). **Honghai Zhang:** Funding acquisition (lead); Project administration (equal).

## Supporting information

Appendix S1

Appendix S2

## Data Availability

All the mitochondria genome sequences used in this study were accessed through the GenBank database using the accession numbers and DOI accession numbers in Appendix S1.
